# Cationized gelatin-HVJ envelope with sodium borocaptate improved the BNCT efficacy for liver tumors *in vivo*

**DOI:** 10.1186/1748-717X-6-8

**Published:** 2011-01-20

**Authors:** Hitoshi Fujii, Akifumi Matsuyama, Hiroshi Komoda, Masao Sasai, Minoru Suzuki, Tomoyuki Asano, Yuichiro Doki, Mitsunori Kirihata, Koji Ono, Yasuhiko Tabata, Yasufumi Kaneda, Yoshiki Sawa, Chun Man Lee

**Affiliations:** 1 Department of Surgery, Osaka University Graduate School of Medicine, Osaka, Japan; 2 Medical Center for Translational Research, Osaka University Hospital, Osaka, Japan; 3 Particle Radiation Oncology Research Center Laboratory, Research Reactor Institute, Kyoto University, Osaka, Japan; 4 Department of Agriculture, Osaka Prefectural University, Osaka, Japan; 5 Department of Biomaterials, Institute for Frontier Medical Sciences, Kyoto University, Kyoto, Japan; 6 Division of Gene Therapy Science, Osaka University Graduate School of Medicine, Osaka, Japan; 7 Health Care Economics and Industrial Policy, Osaka University Graduate School of Medicine, Osaka Japan

## Abstract

**Background:**

Boron neutron capture therapy (BNCT) is a cell-selective radiation therapy that uses the alpha particles and lithium nuclei produced by the boron neutron capture reaction. BNCT is a relatively safe tool for treating multiple or diffuse malignant tumors with little injury to normal tissue. The success or failure of BNCT depends upon the ^10^B compound accumulation within tumor cells and the proximity of the tumor cells to the body surface. To extend the therapeutic use of BNCT from surface tumors to visceral tumors will require ^10^B compounds that accumulate strongly in tumor cells without significant accumulation in normal cells, and an appropriate delivery method for deeper tissues.

Hemagglutinating Virus of Japan Envelope (HVJ-E) is used as a vehicle for gene delivery because of its high ability to fuse with cells. However, its strong hemagglutination activity makes HVJ-E unsuitable for systemic administration.

In this study, we developed a novel vector for ^10^B (sodium borocaptate: BSH) delivery using HVJ-E and cationized gelatin for treating multiple liver tumors with BNCT without severe adverse events.

**Methods:**

We developed cationized gelatin conjugate HVJ-E combined with BSH (CG-HVJ-E-BSH), and evaluated its characteristics (toxicity, affinity for tumor cells, accumulation and retention in tumor cells, boron-carrying capacity to multiple liver tumors *in vivo*, and bio-distribution) and effectiveness in BNCT therapy in a murine model of multiple liver tumors.

**Results:**

CG-HVJ-E reduced hemagglutination activity by half and was significantly less toxic in mice than HVJ-E. Higher ^10^B concentrations in murine osteosarcoma cells (LM8G5) were achieved with CG-HVJ-E-BSH than with BSH. When administered into mice bearing multiple LM8G5 liver tumors, the tumor/normal liver ratios of CG-HVJ-E-BSH were significantly higher than those of BSH for the first 48 hours (*p < 0.05*). In suppressing the spread of tumor cells in mice, BNCT treatment was as effective with CG-HVJ-E-BSH as with BSH containing a 35-fold higher ^10^B dose. Furthermore, CG-HVJ-E-BSH significantly increased the survival time of tumor-bearing mice compared to BSH at a comparable dosage of ^10^B.

**Conclusion:**

CG-HVJ-E-BSH is a promising strategy for the BNCT treatment of visceral tumors without severe adverse events to surrounding normal tissues.

## Background

Boron neutron capture therapy (BNCT) is a cell-selective radiation therapy that uses alpha particles and lithium nuclei produced by the boron neutron capture reaction. These particles cause cell destruction, bouncing out to a maximum distance of 10 μm from the target, a distance that corresponds to the size of a cell. These particles only destroy the cells that take up ^10^Boron (^10^B) [1]. This therapy is clinically indicated for multiple and diffuse tumors, such as glioblastoma and head and neck tumors [2]. BNCT was recently evaluated for treating liver tumors [3-8], although the prognosis of patients treated by BNCT with conventional ^10^B compounds, particularly sodium borocaptate (BSH), is not good because of its low accumulation in liver tumors and the attenuation of the epithermal neutron beams directed toward deep lesions [9-11]. Therefore, treating liver tumors effectively with BNCT will require novel ways of delivering BSH, with the characteristics of high accumulation in the tumor, low toxicity for normal tissue, and rapid withdrawal from normal tissue and the bloodstream [12]. Various carriers such as liposomes have been investigated [13-16], but until now a vector for BSH that adequately satisfies the above requirements has not been developed.

Liver tumors, including primary and secondary tumors, are the fifth most common solid tumor worldwide. The incidence is increasing rapidly in most countries, at a pace that will make liver tumors the third most common tumor by 2030 [17,18]. The mortality rate of liver tumors, especially multiple metastatic liver tumors, is high. Multimodal therapies for multiple liver tumors have advanced considerably, and include radiofrequency ablation, radiation, surgical extirpation and transplantation [19]. However, therapy for multiple and diffuse liver tumors is still difficult, because reducing the liver volume reduces its organ function. Therefore, a therapy selective for tumors with minimal damage to normal liver tissue is of great interest.

Hemagglutinating Virus of Japan Envelope (HVJ-E) is a simple vector that is converted into an inactivated virus containing lipid envelope for gene transfer vector originally [20]. HVJ-E has been used to carry anticancer drugs with some success [21,22]. HVJ-E is reported both to possess high fusion ability and to elicit anti-tumor immune responses [23,24], making it an attractive candidate for widespread use in cancer therapy. On the other hand, HVJ-E has strong hemagglutination activity, making it unsuitable to administer systemically. There are no reports describing the systemic administration of HVJ-E in cancer therapy, although one study reports improved HVJ-E stability in the bloodstream when it is administered with a cationized gelatin [25]. The development of a novel HVJ-E-based vector that can be administered into the general circulation is highly desirable for cancer treatment.

We therefore focused on HVJ-E because of its versatility, its high fusion ability, and its ability to stimulate an immune response. We developed a cationized gelatin conjugate of HVJ-E with BSH that can be administered into the general circulation, and we evaluated its safety, bio-distribution, and effectiveness in BNCT treatment using a murine model of multiple liver tumors.

## Materials and methods

### Mice

Female C3H/HeN Jcl mice at 8-12 weeks of age were obtained from CLEA Japan (Tokyo, Japan) and kept in standard housing. Body weight of mice was 19.6 ± 1.6 (17-23) g at each experiment. All animal experiments were performed under a protocol approved by the Ethics Review Committee for Animal Experimentation of Osaka University Graduate School of Medicine.

### Cell line

The cell line of murine osteosarcoma (LM8G5), which was isolated from LM8 cells after five successive cycles of *in vivo *selection procedures, were used because of their high potential for metastasizing to the liver [26,27]. The cells were maintained in D-MEM (Sigma Aldrich Japan, Tokyo, Japan) containing 10% fetal bovine serum, 1% (v/v) 100 × non-essential amino acids, 1 mM sodium pyruvate, 2 mM L-glutamine, 50 μM 2-mercaptoethanol, 100 units/ml penicillin, and 100 μg/ml streptomycin.

### Animal Model

LM8G5 cells (1 × 10^6 ^cells in 200 μl, with serum-free medium) were injected into the surgically exposed ileocolic vein of mice under general anesthesia with Avertin (2.5% tribromoethanol at a concentration of 1 ml/100 g live weight). Multiple small liver tumors were observed seven days after the injection by exploratory laparotomy, and these tumors led to the death of the mice within 20 days after tumor inoculation.

### HVJ-E

HVJ was purified from chicken egg chorioallantoic fluid by centrifugation, and the titer calculated as previously described [20]. The virus was inactivated by UV irradiation exposure (99 mJ/cm^2^) just before use, eliminating the ability of the virus to replicate while leaving its fusion capacity intact, as previously described [20].

### Cationized Gelatin (CG) and BSH

Gelatin was prepared from pig skin type I collagen through an acid process, and was kindly supplied by Nitta Gelatin (Osaka, Japan). Ethylenediamine (ED), glutaraldehyde, 2,4,6-trinitrobenzenesulfonic acid, β-alanine, and a protein assay kit (# L8900) were purchased from Nacalai Tesque (Kyoto, Japan). The coupling agent, 1-ethyl-3-(3-dimethylaminopropyl) carbodiimide hydrochloride salt (EDC), was obtained from Dojindo Laboratories (Kumamoto, Japan). The CG was prepared by introducing ED to the carboxyl groups of low-molecular-weight gelatin (M.W. 3,100), as previously described [28]. Sodium borocaptate (Na_2 _^10^B_12_H_11_SH: BSH), was obtained from Stella Chemifa (Osaka, Japan).

### Incorporation into HVJ-E

To incorporate plasmid DNA or BSH into HVJ-E, 10 μl of HVJ-E suspension (1.0 × 10^10 ^particles) was added to 15 μl of 1% protamine sulfate, and this was mixed with plasmid DNA (200 μg) or BSH (6,667 μg boron) and 40 μl of 3% Tween-80 diluted with TE solution (10 mM Tris-HCl, pH 8.0, 1 mM EDTA). Qdot 655 ITK Carboxyl Quantum Dots (Qdot; Invitrogen, Carlsbad, CA, USA) were introduced into HVJ-E by electroporation (250 V, 750 μF). The mixture was centrifuged at 15,000 rpm for 15 min at 4°C. To remove the detergent and unincorporated plasmid DNA, BSH, or Qdot, the pellet was washed with 1 ml of balanced salt solution (10 mM Tris-HCl, pH 7.5, 137 mM NaCl, and 5.4 mM KCl), and the envelope vector was suspended in 1,000 μl of phosphate-buffered saline (PBS). To determine the ^10^B concentration in the HVJ-E combined with BSH, the complex was digested with nitric acid solution at Bio Research (Hyogo, Japan) and assayed with inductively coupled plasma-atomic emission spectrometry (ICP-AES, ULTIMA2, Horiba Jobin Yvon, Kyoto, Japan).

### Cationized Gelatin conjugate HVJ-E (CG-HVJ-E)

The CG-HVJ-E complex was formed by mixing the two materials in an aqueous solution. Briefly, 750 μg of CG was added to 150 μl of 0.1 M PBS (pH 7.4) containing 4.5 × 10^9 ^particles of HVJ-E. The solution was mixed by tapping several times. The solution was then incubated on ice for 15 min to form CG-HVJ-E. The CG-HVJ-E vector was purified by centrifugation as described above.

### Zeta potential and particle size of HVJ-E compounds

The zeta potential of each HVJ-E complex (HVJ-E, CG-HVJ-E, HVJ-E-BSH, and CG-HVJ-E-BSH) was measured by an electrophoretic light scattering (ELS) assay (ELS-7000AS, Otsuka Electric Co. Ltd., Osaka, Japan) at 37°C with an electric field strength of 100 V/cm [29]. The particle size of each compound was measured by a dynamic light scattering (DLS) assay (Submicron Particle Analyzer N5, Beckman Coulter, Fullerton, CA, USA).

### Transmission microscopy

Ultra-thin layers of HVJ-E, CG-HVJ-E, and CG-HVJ-E-BSH stained with 3% uranylacetate were examined with an electron microscope (H-7650 and S-800, Hitachi, Tokyo, Japan) to determine the particle size.

### Hemagglutination assay

The hemagglutination assay was done in a 96-well round-bottom plate using 50 μl/well of a 0.5% suspension of chicken red blood cells (Nippon Bio-Test Laboratories, Tokyo, Japan) and 50 μl/well of an HVJ-E solution serially diluted with PBS [30].

### Acute toxicity in normal mice

Each HVJ-E complex was administered by intra-cardiac injection (200 μl) into 8-12-week-old female C3H/HeN mice, which were monitored for 7 days for survival.

### Blood chemistry monitoring after systemic administration of HVJ-E complexes

Indications of systemic injury were recorded, including serum levels of total bilirubin (T. Bil), aspartate aminotransferase (AST), and alanine aminotransferase (ALT) as markers of liver function, lactate dehydrogenase (LDH) and blood urea nitrogen (BUN) as markers of hemagglutination, and creatinine (Cr) as a marker of renal function. All marker levels were measured using an automated analyzing system (BML, Tokyo, Japan) at 24 and 48 hours and at 7 days after systemic administration of 4.5 × 10^9 ^HVJ-E particles.

### Affinity of HVJ-E complexes to tumor cells and localization of Qdot carried in HVJ-E complexes

HVJ-E (1.5 × 10^9 ^particles) and CG (250 μg) were combined to produce CG-HVJ-E. LM8G5 cells (2 × 10^4^) were seeded into each well of an 8-well Lab-tek chamber (Nalge Nunc International, Rochester, NY, USA) and cultured overnight. The cells were incubated with Qdot alone or Qdot with HVJ-E or CG-HVJ-E, at a concentration of 2.5 × 10^8 ^Qdot particles per well for 1 hour. The cells were washed twice with PBS and fixed with 4% paraformaldehyde. Hoechst 33342 (10 μM, Invitrogen) was used to stain the nuclei, and the cells were viewed with fluorescence microscopy (BX61, Olympus, Tokyo, Japan). To visualize the intracellular localization of the Qdot carried in the HVJ-E or CG-HVJ-E, the cells were stained with Hoechst 33342 for the nucleus and Alexa Fluor 488 phalloidin (Invitrogen) for the cytoplasm, and were viewed by confocal microscopy (Fluoview FV1000, Olympus).

### Transfection efficiency of HVJ-E complexes into tumor cells

The various HVJ-E complexes were incubated with tumor cells to evaluate their transfection efficiency. LM8G5 cells (2 × 10^4^) were seeded into each well of a 96-well plate, cultured overnight with 200 μl of culture medium, and washed with PBS. Each HVJ-E complex with or without luciferase-expressing plasmids (50 μl; 1.5 × 10^9 ^particles) was incubated with tumor cells for 30 min, and then incubated for 30 min at 37°C. After washing twice with PBS, the cells were incubated with fresh medium for 24 hours and then lysed with Lysis Buffer (Promega, Madison, WI, USA). Luciferase activity in the cells was then measured with a Luciferase Assay kit (Promega) using a fluorescence plate reader (Mithras LB 940, Berthold Technologies, Bad Wildbad, Germany). The protein content of the samples was assayed by the Bradford method [31].

### Accumulation and retention of BSH or CG-HVJ-E-BSH in tumor cells *in vitro*

Tumor cells of the LM8G5 cell line (1 × 10^6^) were seeded in 75 cm^2 ^tissue culture flasks and were cultured overnight. The cells were then washed with PBS, 1 ml of BSH (20 μg boron/ml) or CG-HVJ-E-BSH (20 μg boron/ml) was added to each flask, and the mixture was incubated for 30 min at 37°C. The cells were then washed twice with PBS, and the ^10^B concentration in the cells was immediately measured by ICP-AES (Horiba Jobin Yvon) as the initial ^10^B value bound to the cells. Other flasks were incubated an additional 24-48 hours at 37°C and the cells were double-washed again before being tested for ^10^B concentration, which was measured as the ^10^B value.

### Bio-distribution of BSH or CG-HVJ-E-BSH in normal or liver tumor-bearing mice

Mice were injected with 200 μl of BSH (35 μg boron/g ) or 200 μl of CG-HVJ-E-BSH (1.2 μg boron/g ), administered into the general circulation. At 1, 24, or 48 hours after the injection, mice were sacrificed and peripheral blood samples collected. The lung, liver, kidney and spleen were removed after whole-body perfusion with heparinized saline, and weighted. The extracted tissues were digested with the M-Per mammalian protein extraction reagent (Pierce Chemical Co., Rockford, IL, USA) and ultrasonic homogenizer (H3-350, Kawajiri Machinery, Hyogo, Japan), and the ^10^B concentration in each sample was measured by ICP-AES (Horiba Jobin Yvon). The ^10^B accumulation into each organ was calculated as the percentage of ^10^B per weight of each organ.

### Neutron capture autoradiography imaging of murine liver sections after BSH or CG-HVJ-E-BSH administration

Mice bearing liver tumors were given either 35 μg boron/g of BSH or 1.2 μg boron/g of CG-HVJ-E-BSH, administered into the general circulation. The mice were sacrificed 1 hour after BSH administration or 24 hours after CG-HVJ-E-BSH administration. The liver was removed after whole-body perfusion with heparinized saline. Frozen 16-μm-thick liver sections were mounted on Baryotrak-P detector plates (Nagase-Landauer, Tokyo, Japan) and air-dried for 60 min. The samples were exposed to thermal neutrons at a rate of 2.1 × 10^13 ^neutrons/m^2^·s^1 ^for 1 hour at the Japan Research Reactor 4 (JRR-4). For α-auto-radiographic imaging, the detector plates were etched in 7 N NaOH at 70°C for 2 hours to reveal the proton tracks produced by the boron neutron capture reaction [32]. The number of α particles per 10,000 μm^2 ^in each section was counted using VH Analyzer software (Biozero, Keyence, Osaka, Japan).

### Antitumor efficacy of BNCT for murine liver tumors with BSH or CG-HVJ-E-BSH

Mice bearing liver tumors were irradiated with a thermal neutron beam at the JRR-4 8 days after tumor cell inoculation. The mice were given 1.2 μg boron/g of CG-HVJ-E-BSH 24 hours before irradiation treatment, or 35 μg boron/g of BSH 1 hour before irradiation treatment, administered into the general circulation. The mice were then set the acrylic stand, and irradiated for 17 min at the Japan Research Reactor 4 (JRR-4). Neutron irradiation was performed in a single fraction using an thermal beam mode I of JRR-4. In the in-air beam characteristics, thermal neutron flux and the γ-ray absorbed dose were 2.1 × 10^13 ^neutrons/m^2^·s^1 ^and 3.6 Sv/h at a reactor power of 3.5 MW, respectively. To evaluate the effect of BNCT treatment on the liver tumors, the mice were sacrificed 6 days after irradiation, and the livers removed, weighed, and evaluated for pathologic changes. In a separate experiment, 1.2 μg boron/g of BSH or 1.3 μg boron/g of CG-HVJ-E-BSH was administered, the mice were either irradiated or not, and their survival time after irradiation was recorded.

### Statistical analyses

Student's *t*-test was used to determine whether the differences between the various groups were significant. Differences between groups in the survival experiment were determined using the Kaplan-Meier log-rank test. A *p*-value of less than 0.05 was considered statistically significant.

## Results

### CG-HVJ-E characteristics

SDS-PAGE results confirmed that when mixed and centrifuged with HVJ-E, the CG bound to HVJ-E in a dose-dependent manner within a certain range (data not shown). The optimal ratio of CG to HVJ-E, in which the CG-HVJ-E containing luciferase plasmid was transferred most efficiently into LM8G5 cells (data not shown), was identified as 1 μg to 6.0 × 10^6 ^particles, and the zeta potential and particle size of the resulting CG-HVJ-E conjugate was measured (Table 1). CG- HVJ-E was more positive (-14.7 mV) than HVJ-E (-25.1 mV). The form and size of these particles were estimated by using Transmission Electron Microscopy (TEM) and Scanning Electron Microscopy (SEM). HVJ-E, CG-HVJ-E, and CG-HVJ-E-BSH were approximately 300, 300, and 500 nm in diameter, respectively, as measured by TEM (Additional file 1, Figure S1). The DLS assay results were similar (data not shown). Therefore, these data are able to give an estimate that incorporating BSH into the HVJ-E complexes made them larger and slightly more positive than either HVJ-E or CG-HVJ-E.

**Table 1:** Zeta potential and particle sizes of each HVJ-E complex

Complex	Apparent molecular size (nm)	Zeta potential (mV)
HVJ-E	293 ± 32	-25 ± 1
CG-HVJ-E	297 ± 21	-15 ± 3
HVJ-E-BSH	448 ± 144	-28 ± 1
CG-HVJ-E-BSH	494 ± 196	-19 ± 2

### CG-HVJ-E had less hemagglutination activity in vitro and was less toxic than HVJ-E in mice

**Figure 1:**
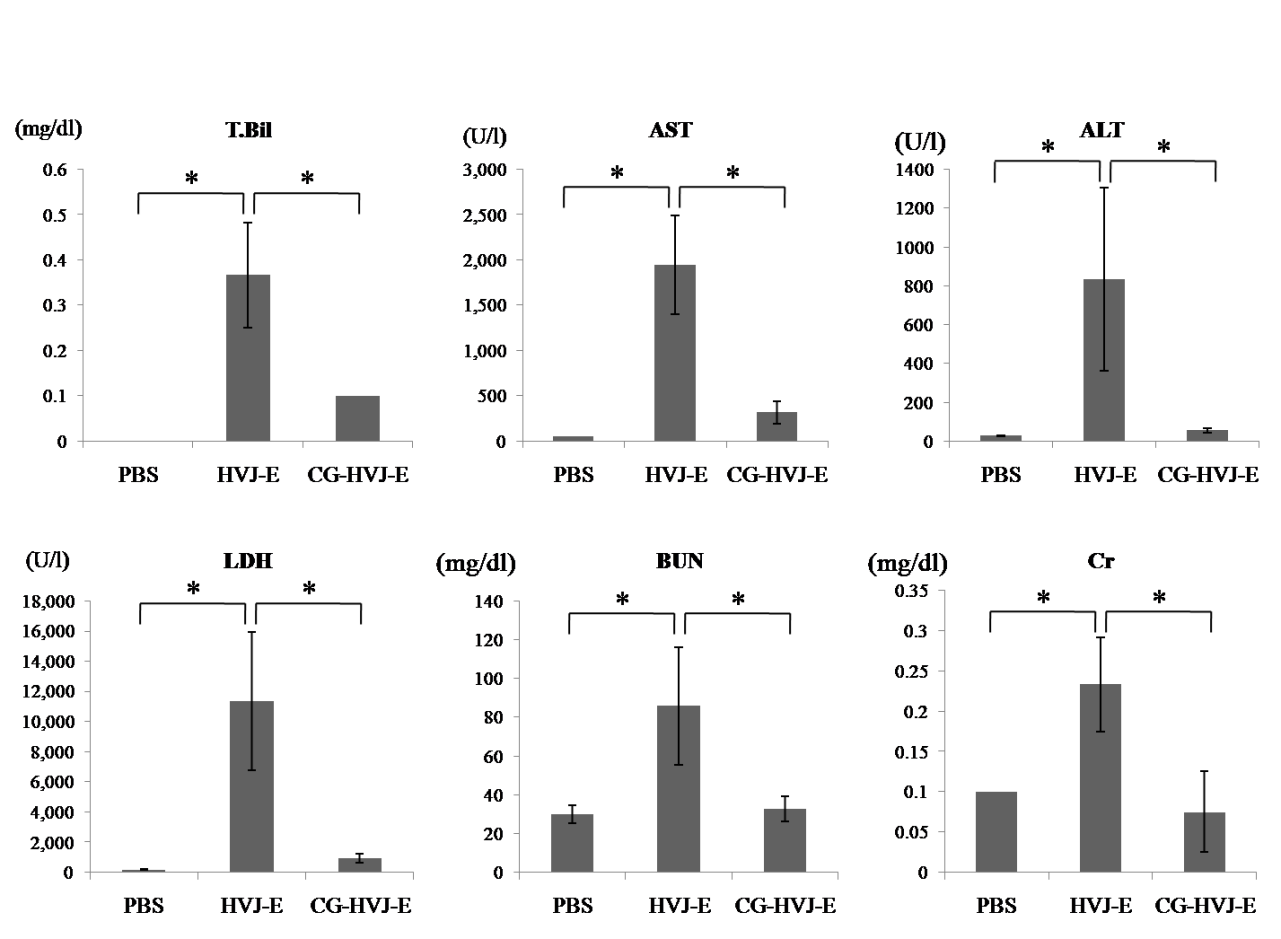
**Blood chemistry tests 24 hours after HVJ-E and CG-HVJ-E administration into normal mice**. Blood markers (T.Bil, AST, ALT, LDH, BUN, and Cr) in normal mice tested 24 hours after intra-cardiac injection of PBS, HVJ-E or CG-HVJ-E. * *p *< 0.05. Results shown are the means ± SD (n = 4).

Hemagglutination is caused by hemagglutinin-neuramidase (HN) protein on the HVJ-E membrane [33]. The hemagglutination of chicken blood cells by CG-HVJ-E was approximately half that of HVJ-E (data not shown). The acute toxicity was determined by administering various concentrations of HVJ-E or CG-HVJ-E to normal mice and monitoring their survival over 7 days; the 100% survival dosage of CG-HVJ-E (6.0 × 10^9 ^particles) was higher than that of HVJ-E (4.5 × 10^9 ^particles). Blood tests done 24 hours after the administration of 4.5 × 10^9 ^particles of HVJ-E or 4.5 × 10^9 ^particles of CG-HVJ-E showed that blood chemistry markers in the CG-HVJ-E-treated mice were almost within the normal range, while those in the HVJ-E-treated mice were significantly higher (Figure 1). These levels peaked 24 hours after administration in mice treated with HVJ-E, and became normal at 7 days (data not shown).

### High affinity and infusion ability of CG-HVJ-E in tumor cells

**Figure 2:**
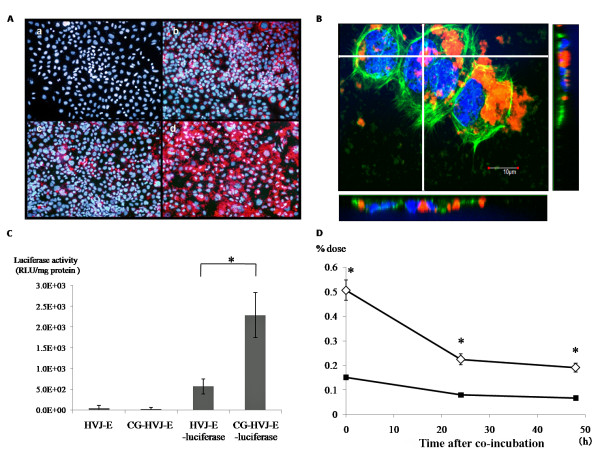
**Affinity of CG-HVJ-E for tumor cells and the intracellular uptake of molecules incorporated into HVJ-E**. A) Affinity of HVJ-E and CG-HVJ-E for tumor cells. LM8G5 cells were incubated alone (a), or with Qdot (b), HVJ-E-Qdot (c), or CG-HVJ-E-Qdot (d) for 60 min in a Lab-tek chamber slide and examined for Qdot (red) and Hoechst 33342 (blue) by fluorescence microscopy, Representative views are shown. B) Intracellular localization of Qdot transported by CG-HVJ-E. Tumor cells were incubated with CG-HVJ-E-Qdot (orange) and stained with Hoechst 33342 (blue) and Alexa Fluor 488 phalloidin (green). Image shows 3-dimensional analysis with confocal microscopy. C) Luciferase activity in tumor cells transfected with HVJ-E or CG-HVJ-E. Cells were cultured for 30 min with HVJ-E or CG-HVJ-E containing a luciferase-expressing plasmid. Luciferase activity was measured 24 hours later to evaluate the transfection efficiency. Results are shown as means ± SD (n = 4). Similar results were obtained in three experiments. * *p *< 0.05. D) ^10^B accumulation and retention in tumor cells *in vitro*. Cells were incubated with 20 μg boron/ml of BSH or CG-HVJ-E-BSH for 30 min, then washed twice with PBS, and the ^10^B concentration was measured by ICP-AES. Separately, cells were incubated in the same manner, but after washing, were incubated in medium without BSH for 24 or 48 hours before testing for ^10^B concentration as described above. The horizontal axis shows time after co-incubation. The vertical axis shows the percent of the administered dose (% dose) of CG-HVJ-E-BSH (open diamond) or BSH (solid square). Results shown are the means ± S.D. (n = 3). * *p *< 0.05.

CG-HVJ-E containing Qdot had a higher affinity for tumor cells than Qdot alone or HVJ-E containing Qdot (Figure 2A). CG-HVJ-E containing Qdot was taken into the cytoplasm, and some Qdots were localized to the nuclei, as seen by confocal microscopy (Figure 2B).

### CG-HVJ-E transfection into tumor cells *in vitro *was highly efficient

CG-HVJ-E's *in vitro *transfection efficiency into tumor cells was 4 times greater than that of HVJ-E, as assessed by a luciferase assay, and it was not cytotoxic (Figure 2C). The enhanced transfection efficiency of CG-HVJ-E was also observed in another tumor cell line (CT26: murine colon cancer, data not shown).

### CG-HVJ-E-BSH increased ^10^B accumulation and retention in tumor cells *in vitro *compared to BSH

The concentration of ^10^B was significantly higher in cells incubated with CG-HVJ-E-BSH than in those incubated with BSH (*p < 0.05*). The ^10^B levels gradually decreased in both cell groups, but the levels were significantly higher in the cells incubated with CG-HVJ-E-BSH than in those with BSH for at least 48 hours after incubation (Figure 2D). These results indicate that CG-HVJ-E-BSH binds rapidly to tumor cells and that the ^10^B contained in CG-HVJ-E-BSH is internalized into the cytoplasm or the nucleus. Adding CG-HVJ-E-BSH to tumor cells *in vitro *resulted in sufficient ^10^B accumulation and retention in the cells to be useful for BNCT.

### BSH incorporated into CG-HVJ-E accumulated in liver tumors and rapidly disappeared from normal tissues in tumor-bearing mice

**Figure 3:**
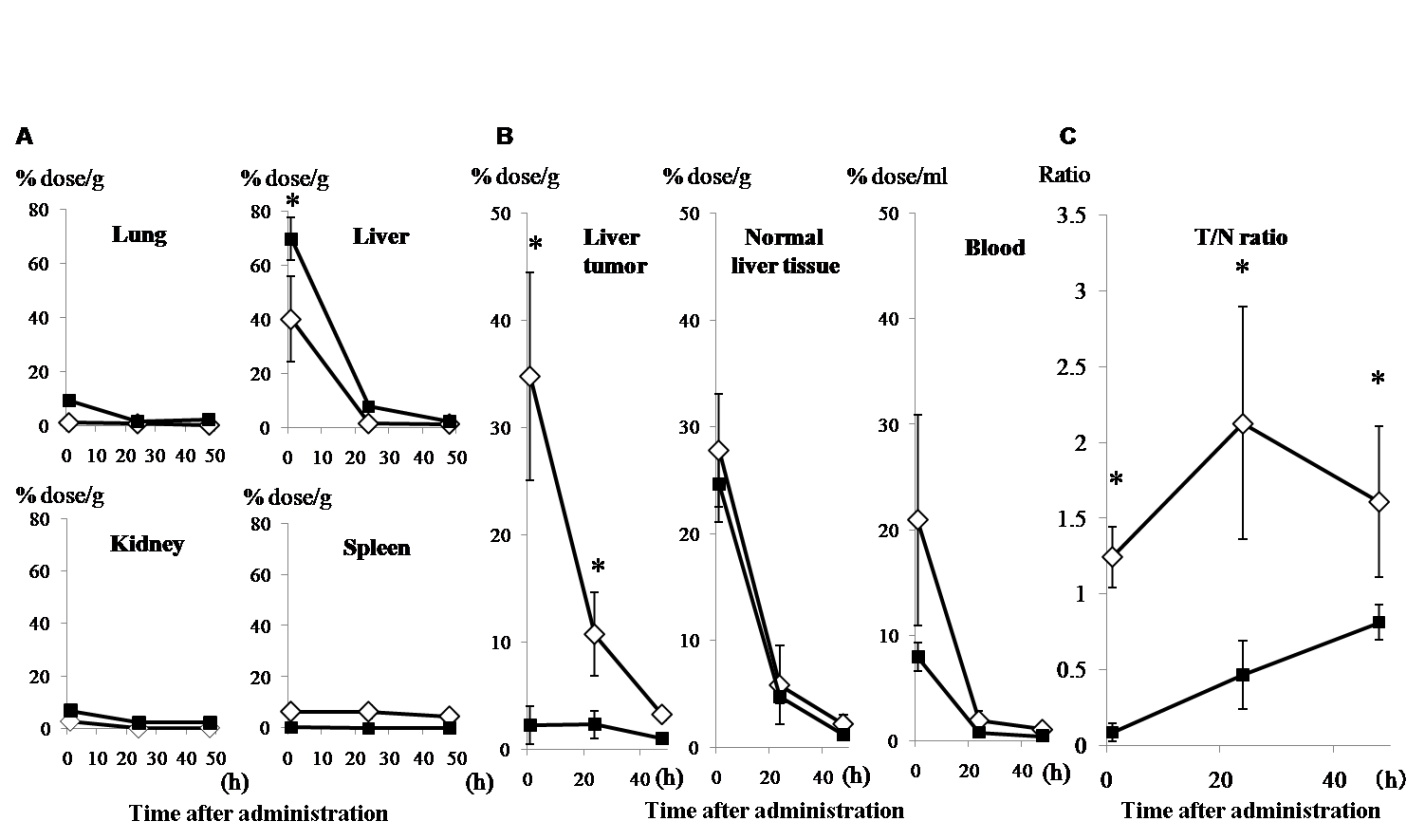
**Bio-distribution of ^10^B in mice with normal liver or with liver tumors**. A) Time course of organ (lung, liver, kidney, and spleen) uptake of ^10^B delivered by 1.2 μg boron/g of BSH or CG-HVJ-E-BSH in normal mice. B) Time course of tumor accumulation (left panel), liver uptake (middle panel), and blood residence (right panel) of ^10^B delivered by 1.2 μg boron/g of BSH or CG-HVJ-E-BSH in tumor-bearing mice. The horizontal axis shows the time after administration. The vertical axis shows the percent of the administered dose per gram of tissue (% dose/g). C) Time course of the Tumor-to-Normal liver tissue (T/N) ^10^B concentration ratio for CG-HVJ-E-BSH (open diamond) or BSH solution (solid square). Data are expressed as the mean ± S.D. (n = 3). * *p <*0.05 compared with BSH.

In normal mice, the ^10^B concentration in the liver 1 hour after administration was higher with BSH than with CG-HVJ-E-BSH. The concentration of both compounds started to decrease by 48 hours after administration. The ^10^B concentration in the lung, kidney, and spleen was low at all time points with both compounds (Figure 3A). In the liver tumor model, BSH and CG-HVJ-E-BSH behaved similarly in the normal liver tissue surrounding the tumors (Figure 3B, middle panel). In the tumors, however, the concentration of ^10^B at 1 and 24 hours after administration was significantly higher with CG-HVJ-E-BSH (34.76 and 10.71% dose/g) than with BSH (2.21 and 2.29% dose/g) (Figure 3B, left panel). In the bloodstream, the ^10^B concentration at 1 hour after administration tended to be higher with CG-HVJ-E-BSH (20.9% dose/ml) than with BSH (7.96% dose/ml), despite the lower quantity of ^10^B administered with both boron compounds (1.2 μg boron/g). From 24 hours after administration and onward, the concentration of ^10^B from both compounds was the same (Figure 3B, right panel).

### Tumor/Normal liver ^10^B ratio in murine liver tumors was greater with CG-HVJ-E-BSH

The Tumor/Normal (T/N) liver ^10^B ratio with CG-HVJ-E-BSH was significantly higher than with BSH from 1 to 48 hours after administration (*p *< 0.05), with a peak difference at 24 hours (p < 0.05; Figure 3C). The Tumor/Blood ^10^B ratio of CG-HVJ-E-BSH also remained higher than that of BSH from 1 to 48 hours after administration (data not shown).

### CG-HVJ-E-BSH improved the T/N ^10^B ratio in neutron capture autoradiography images of murine liver tumors

**Figure 4:**
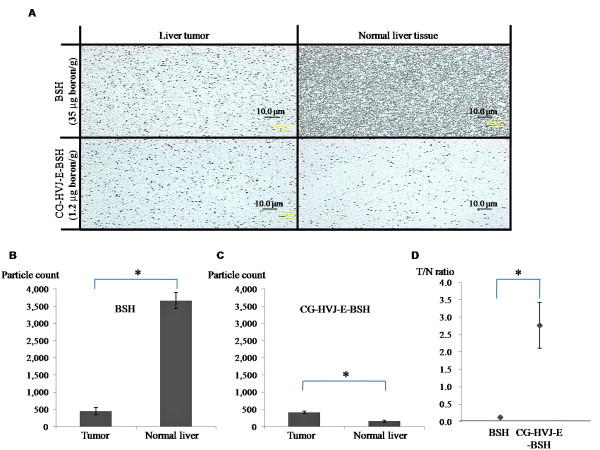
**Neutron capture radiographic image in murine liver sections after administration of BSH or CG-HVJ-E-BSH**. Liver sections from tumor-bearing mice were prepared and frozen 1 hour after BSH (35 μg boron/g) injection or 24 hours after CG-HVJ-E-BSH (1.2 μg boron/g) injection. The sections were placed on CR-39 detector plates and exposed to thermal neutrons (2.1 × 10^13 ^neutrons/m^2^·s^1^) for 1 hour. A) The number of α particles per 10,000 μm^2 ^section was counted by VH Analyzer software after NaOH etching. B) The number of α particles per 10,000 μm^2 ^section of BSH or C) of CG-HVJ-E-BSH (n = 3). D) Tumor-to-normal liver tissue (T/N) ratio for the number of α particles

Neutron capture autoradiography (NCAR) was performed after BSH (35 μg boron/g) or CG-HVJ-E-BSH (1.2 μg boron/g) was injected into mice bearing liver tumors. The ^10^B particle count in the BSH- and CG-HVJ-E-BSH-treated livers are shown in Figure 4B and 4C. The T/N ratio 1 hour after BSH administration was 0.12, and that for CG-HVJ-E-BSH at 24 hours after administration was 2.76 (Figure 4D), which is similar to the values obtained in the bio-distribution study. It is of interest that the T/N ^10^B ratio was higher with CG-HVJ-E-BSH, even though the actual quantity of ^10^B was 30 times greater in the BSH dosage. The number of α particles with CG-HVJ-E (415 ± 35) was similar to that of BSH (451 ± 107) in the liver tumor sections (Figure 4A).

### BNCT with CG-HVJ-E-BSH inhibited tumor growth, preserved the normal surrounding liver tissue, and prolonged survival time in the murine liver tumor model

To evaluate the use of BNCT with CG-HVJ-E-BSH for murine liver tumors, BNCT was performed on mice bearing LM8G5 liver tumors. To assess the T/N ratio of CG-HVJ-E-BSH, BNCT was performed 24 hours after CG-HVJ-E-BSH administration or 1 hour after BSH administration [2,4]. We first evaluated the anti-tumor efficacy at 14 days after tumor cell inoculation, because up to that time, the tumor-bearing mice were severely damaged by the radical spread of tumors (about 50% of the untreated mice were dead). Therefore, we sacrificed the tumor-bearing mice that were alive until that time to evaluate the efficacy of BNCT.

**Figure 5:**
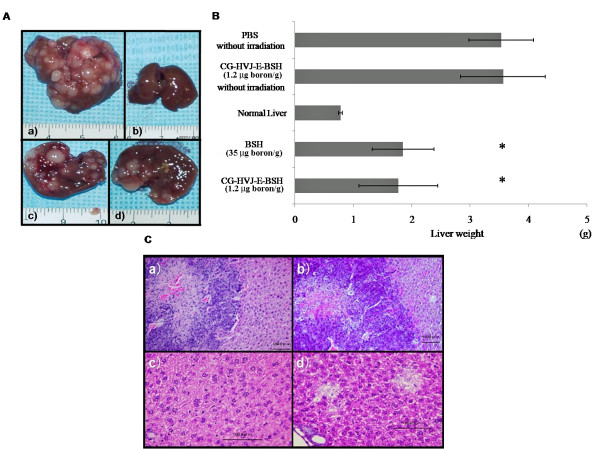
**Anti-tumor efficacy of BNCT in mice with liver tumors**. C3H/HeN mice were given an intra-portal injection of LM8G5 cells (1 × 10^6 ^cells) on day 0. Mice were given a single intra-cardiac injection of CG-HVJ-E-BSH (1.2 μg boron/g) 24 hours before irradiation, or BSH (35 μg boron/g) 1 hour before irradiation. PBS and CG-HVJ-E-BSH solution were administered without neutron irradiation as a control. After irradiation on day 8, mice were sacrificed on day 14 to determine the BNCT efficacy on tumor metastasis. A) Macroscopic views (a) of the liver with tumors after administration of PBS; (b) normal liver; (c) liver with tumors after BNCT with BSH, and (d) liver with tumors after BNCT with CG-HVJ-E. B) Liver weight after BNCT. * *p *< 0.05 compared with PBS or CG-HVJ-E without irradiation. (each group n = 4). C) Representative light microscopic views of liver tumor tissue (upper panels, low magnification) or normal liver tissue (lower panels, high magnification) 6 days after BNCT with CG-HVJ-E-BSH (1.2 μg boron/g) (a, c) or BSH (35 μg boron/g) (b, d). Sections are stained with hematoxylin-eosin. Bar: 100 μm.

BNCT with CG-HVJ-E-BSH (1.2 μg boron/g) inhibited the local growth of liver metastases as much as BNCT with BSH (35 μg boron/g). This dosage of BSH was determined from the clinical dose for BNCT for various malignant tumors, and effectively contained 35 times the ^10^B that was present in the CG-HVJ-E-BSH dosage (Figure 5A, B). Some histological damage, which appeared, for example as fractionated or vacuolated cells, was observed in both the tumor mass and in the normal liver tissue after BNCT with BSH (35 μg boron/g) (Figure 5C-b, d). In contrast, little histological damage was detected in the normal liver tissue surrounding the tumors after BNCT with CG-HVJ-E-BSH (Figure 5C-a, d). We originally thought that the damage to the liver might have been influenced by the longer survival time of mice treated with BSH and BNCT; however, the survival rate of these mice at 14 days after tumor cell inoculation was 37.5% (Additional file 2, Figure S2). This survival time was shorter than that of the untreated tumor-bearing mice. As we were not able to be certain if this dosage of BSH was a clinical equivalent, we used a dose of 1.3 μg boron/g of BSH to evaluate the survival time after BNCT, compared to a dose of 1.2 μg boron/g of CG-HVJ-E-BSH.

Finally, we compared the effectiveness of BNCT against tumors when used with BSH or CG-HVJ-E-BSH, in terms of survival after BNCT. With the assumption that the survival time of tumor-bearing mice after BNCT with a high dose of BSH (35 μg boron/g) was affected by normal liver damage as well as anti-tumor efficacy, both compounds were administered at dosages with similar ^10^B concentrations (CG-HVJ-E-BSH, 1.2 μg boron/g or BSH,1.3 μg boron/g) into mice bearing liver tumors at 24 hours or 1 hour before irradiation, respectively. Irradiation was performed 8 days after the tumor cell inoculation, and the survival of the mice assessed. CG-HVJ-E-BSH was most effective in increasing the mean survival time of mice bearing liver tumors compared with the other groups (p < 0.005; Additional file 2, Figure S2). We observed little histological damage in the normal liver tissues 6 days after BNCT with the lower dose of BSH (1.3 μg boron/g ) besides the damage that was already present in the tumor mass (Additional file 3, Figure S3).

## Discussion

With the goal of creating a novel BSH vector for effective BNCT, we chose HVJ-E because of its strong fusion ability, its effectiveness as a vehicle for delivering various drugs and genes, and its ability to stimulate an immune response against tumors in local cancer therapy [23]. Clinical trials of locally administered HVJ-E for patients with advanced malignant melanoma are underway in Japan. Although HVJ-E is not suitable for systemic administration because of its strong hemagglutinating activity, it has been reported that combining HVJ-E with 5,000-kDa cationized gelatin greatly improves its stability in the bloodstream [25]. In this study, we developed CG-HVJ-E combined with BSH, which can be administered into the general circulation, unlike HVJ-E, and confirmed its bio-distribution.

We compared the safety and efficacy of CG-HVJ-E-BSH in BNCT with that of BSH, using a murine model for liver tumors. For systemic administration, we developed a smaller CG-HVJ-E with a lower molecular weight (3,300 kDa) CG compared with the previously used CG-HVJ-E, which had a particle diameter of 777 nm [25]. We found that this CG-HVJ-E could be safely administered systemically in mice, with reduced toxicity and hemagglutination compared to HVJ-E (Figure 1). In the bio-distribution test using normal mice, both BSH and CG-HVJ-E-BSH accumulated in the liver immediately, but almost all of the ^10^B had disappeared from the normal liver 48 hours later (Figure 3A). In liver tumors, however, CG-HVJ-E-BSH accumulation was greater than that of BSH although the boron proceeding from CG-HVJ-E-BSH was 35 times higher than that of BSH (Figure 3B); accordingly, the CG-HVJ-E-BSH T/N ratio was significantly higher than that of BSH in tumor-bearing mice, particularly at 24 hours after administration (Figure 3C). Neutron capture autoradiography revealed a higher T/N ^10^B ratio with CG-HVJ-E-BSH than with BSH 1 hour after administration, despite the 35-fold-higher quantity of ^10^B contained in the BSH dosage (Figure 4).

In our experiments, BNCT was performed 1 hour after BSH administration, because it followed the reported procedure for the clinical use of BNCT for liver tumors [9], and there was little difference between the T/N ratio an hour after administration and the ratio over the next 24 hours (Figure 3C). This was due to the protracted circulating time of CG-HVJ-E-BSH in the bloodstream. Therefore, this complex accumulated in the tumor by the enhanced permeability and retention (EPR) effect [34]. In fact, the particle size of the CG-HVJ-E-BSH was suitable for the EPR effect (Table 1) [35]. Another reason for this finding was that CG-HVJ-E has a high affinity and high fusion ability for tumor cells (Figure 2A, B, C). Although ^10^B was taken up by the tumor cells over time, a large number of CG-HVJ-E-BSH molecules were incorporated into the tumor cells immediately, and high ^10^B concentrations were maintained much longer with CG-HVJ-E-BSH than with BSH (Figure 2D). The mechanism for the preferential affinity of CG-HVJ-E to tumor cells as compared with HVJ-E has not been clarified, but it has been reported that when HVJ-E is conjugated with cationized gelatin, the transfection efficiency improves without a loss of cell fusion ability [25]. Therefore, the efficacy of CG-HVJ-E-BSH was similar to the 35-fold higher dose of ^10^B as BSH for suppressing the spread of tumor cells without normal liver injury (Figure 5A, B, C).

When used in BNCT, the CG-HVJ-E-BSH significantly increased the survival time over BSH at an equivalent ^10^B dosage (Additional file 2, Figure S2). Generally, BSH is rarely transferred into the cytoplasm and, once there, is easily removed [36]. On the other hand, CG-HVJ-E-BSH was highly selective for tumor cells and showed both strong fusion ability and the ability to transfer into the tumor cell nucleus. As a result, CG-HVJ-E-BSH improved the effectiveness of BNCT because the ^10^B was highly concentrated and retained in the nuclei of the tumor cells (Figure 2B, C), where its cytotoxicity was much higher than that of ^10^B bound to the tumor cell surface [14,37,38].

Moreover, HVJ-E has the potential to induce a bystander effect, so that CG-HVJ-E-BSH could be incorporated into vicinal cells through gap junctions. It is possible that BNCT with CG-HVJ-E-BSH induces a synergistic effect, resulting in a greater destruction of vicinal tumor cells than is seen with BNCT with BSH, which induces a bystander effect that generates hereditary abnormalities in vicinal cells [39].

We chose multiple liver tumors as a target for evaluating the effectiveness of BNCT with CG-HVJ-E-BSH, because BNCT for multiple liver tumors has not gained popularity and the T/N ratio needs to be improved for deep-site tumors. In the absence of liver function disorders, the response of multiple liver tumors is thought to be a good indication of BNCT effectiveness. In this report, we treated mice bearing liver tumors with BNCT [27] after establishing the presence of tumors of several millimeters in diameter. This murine model appears to reflect the clinical stage that we targeted. BNCT with BSH is not indicated for multiple liver tumors in clinical settings and is only at the experimental stage [9,10]. BNCT was significantly more effective against liver tumors when used with CG-HVJ-E-BSH than with BSH, and normal liver tissue was not injured. The limited injury to normal liver tissue makes more than one BNCT irradiation possible, which is likely to increase the therapeutic potential. However, in these experiments, only one irradiation was done. With regard to BNCT with BSH for clinical liver tumors at deep sites, the required T/N ^10^B ratio is over 15 [36,40]. Moreover, the human trunk is much thicker than the murine trunk. Therefore, for BNCT with CG-HVJ-E-BSH to become an established, effective clinical procedure, further improvements are needed not only in the drug-delivery system, but also in the vessel-selective delivery [41] because of the attenuation of neutron beams directed toward deep lesions.

Our trial of BNCT for multiple liver tumors at deep sites should forward its development to treat other deep-site tumors, such as pancreatic cancer and malignant mesothelioma [42-44], and further the investigation into BNCT and HVJ-E. However, some problems need to be resolved in future experiments, particularly with regard to improving the incorporation of ^10^B into the HVJ-E.

It has been reported that locally administered HVJ-E induces immuno-responses against tumors [23. 24], and effectively transports antitumor drugs [22,45]. Our experiments included a single administration of HVJ-E, which did not appear to have an anti-tumor effect unless accompanied by irradiation (Figure 5B, Additional file 2, Figure S2). However, the fractionated administration of HVJ-E, as is used for other vaccinations, might be possible. To address the limitations of this novel HVJ-E BSH, investigations into concurrent chemo-radiation therapy, fractionated administration with or without ^10^B, and conjugating with ligands for tumor-specific molecules should be performed.

In summary, we developed a form of CG-HVJ-E that could be administered into the general circulation and had both high tumor selectivity and high retention in tumor cells. This vector, when combined with BSH, improved the efficacy of BNCT for multiple liver tumors *in vivo*. Therefore, CG-HVJ-E holds potential for a drug delivery system with clinical applications for cancer therapy.

## Abbreviations

BNCT: Boron Neutron Capture Therapy; BSH: sodium borocaptate; HVJ-E: Hemagglutinating Virus of Japan Envelope.

## Competing interests

All authors declare there were no actual or potential conflicts of interest in this study.

## Authors' contributions

HF carried out the study, and contributed to the conception of the manuscript and the interpretations of the data. AM, HK, MS, MS, AT, and YT participated in the design of the study. YD, MK, and KO provided some intellectual recommendation. YK and YS provided some intellectual recommendation and reviewed the manuscript. CML conceived of the study, and participated in its design and coordination. All authors read and approved the final manuscript.

## Supplementary Material

Additional file 1 **Figure S1. Transmission electron microscope photographs of HVJ-E complexes**. (A) HVJ-E, (B) CG-HVJ-E, and (C) CG-HVJ-E-BSH. Bar: 200 nm. Click here for file

Additional file 2 **Figure S2. Survival of mice treated with BNCT**. Mice were given a single intra-cardiac injection of CG-HVJ-E-BSH (1.2 μg boron/g) 24 hours before irradiation, or BSH (1.3 μg boron/g) 1 hour before irradiation. PBS and CG-HVJ-E-BSH were administered without irradiation as a control. The mean survival time of the mice that received the BNCT treatment with CG-HVJ-E-BSH was significantly longer than that of the other groups (n = 4). * *p *< 0.005 (PBS without neutron irradiation, 1.3 μg boron/g of BSH with neutron irradiation, 1.2 μg boron/g of CG-HVJ-E-BSH without neutron irradiation vs. 1.2 μg boron/g of CG-HVJ-E-BSH with neutron irradiation). Click here for file

Additional file 3 **Figure S3. Representative light microscopy views of the liver tumor (A) and normal liver tissue (B) 6 days after BNCT with a low dose of BSH (1.3 μg boron/g)**. Tissues were stained with hematoxylin-eosin. Bar: 100 μm. Click here for file
