# Supplementary figures and images for: Cationized gelatin-HVJ envelope with sodium borocaptate improved the BNCT efficacy for liver tumors in vivo
Source: Radiat Oncol. 2011 Jan 20;6:8. doi: 10.1186/1748-717X-6-8 (PMC3035588; doi:10.1186/1748-717X-6-8)

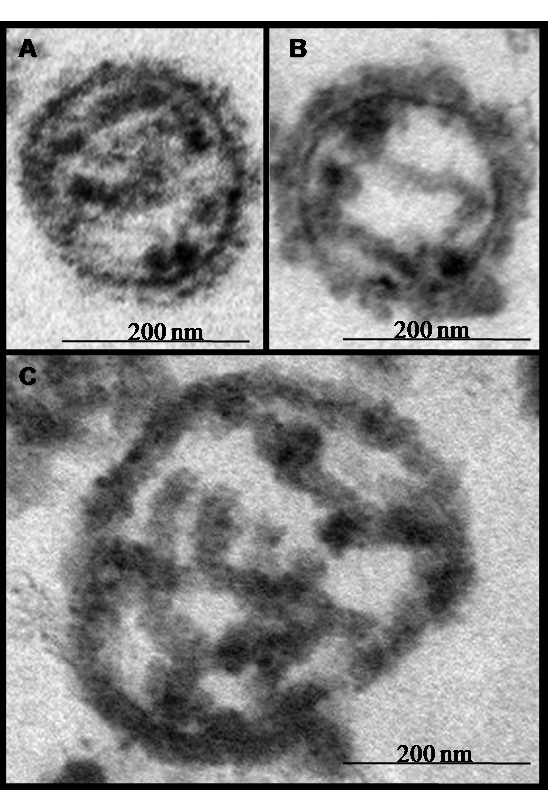

Supplement: Additional file 1 — Figure S1. Transmission electron microscope photographs of HVJ-E complexes. (A) HVJ-E, (B) CG-HVJ-E, and (C) CG-HVJ-E-BSH. Bar: 200 nm. [file 1748-717X-6-8-S1.TIFF]

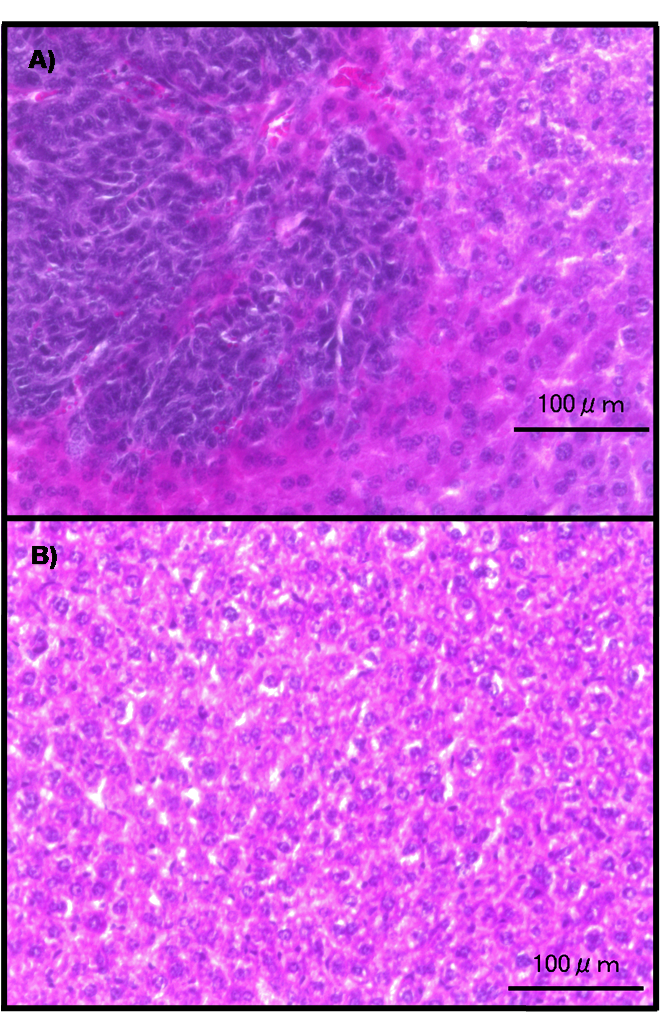

Supplement: Additional file 3 — Figure S3. Representative light microscopy views of the liver tumor (A) and normal liver tissue (B) 6 days after BNCT with a low dose of BSH (1.3 μg boron/g). Tissues were stained with hematoxylin-eosin. Bar: 100 μm. [file 1748-717X-6-8-S3.TIFF]
